# “Medicines is all that I can sometimes offer them”: challenges of providing primary diabetes care to persons with disabilities in Tamil Nadu

**DOI:** 10.1186/s12913-022-08246-1

**Published:** 2022-07-05

**Authors:** Rajeswaran Thiagesan, Hilaria Soundari, Vijayaprasad Gopichandran

**Affiliations:** 1 Centre for Applied Research, The Gandhigram Rural Institute – Deemed to be University, Dindigul District, Tamil Nadu 624 302 Gandhigram, India; 2 Research, Monitoring and Evaluation Lead, Resource Group for Education and Advocacy for Community Health (REACH), Chennai, India; 3 Department of Community Medicine, Employees State Insurance Corporation Medical College and Post Graduate Institute of Medical Education and Research, KK Nagar, 600078 Chennai, India

**Keywords:** Type 2 diabetes, Primary care, Community health worker, Persons with disabilities, Treatment, Lifestyle modification, Health policy

## Abstract

**Background:**

Persons with disabilities have a higher risk for and poorer outcomes of type 2 diabetes. Primary health care providers face several challenges in providing primary diabetes care for them. This study was conducted to explore the challenges faced by primary health care providers in delivering primary diabetes services to persons with disabilities.

**Methods:**

We performed a qualitative research study by conducting in-depth interviews among 13 primary health care providers including medical officers, staff nurses, community health workers and a physiotherapist. We adopted a descriptive qualitative research approach to data collection and analysis.

**Results:**

Primary health care providers often could only prescribe medications to persons with diabetes by proxy due to poor accessibility of the facilities. They felt that these patients also had poor compliance to treatment. They felt that the lack of standard guidelines for diet and exercise for persons with disabilities prevented them from giving them appropriate advice on the same and even if they did, persons with disabilities would find it very difficult to adopt dietary changes and physical activity as they were dependent on others for even their daily activities. They also felt that they couldn’t perform annual screening tests due to lack of accessibility to higher facilities. Some primary care providers did local innovations such as formation of peer support groups, utilization of resources of other programs to reach out to persons with disabilities and innovative physical activity techniques to care for persons with disabilities. They recommended that there is a need for specific guidelines for management of diabetes among persons with disabilities, treatment of chronic diseases among persons with disabilities must be incentivized and there must be intersectoral coordination between social welfare department and health department to achieve the goal of care for persons with disabilities.

**Conclusions:**

Primary health care providers faced substantial challenges in providing primary diabetes care for persons with disabilities. There is a need for an effective public health policy to address these challenges.

**Supplementary Information:**

The online version contains supplementary material available at 10.1186/s12913-022-08246-1.

## Background

Type 2 diabetes is a major global public health problem [1]. It affects 415 million people worldwide and is expected to increase to 640 million by 2040. The prevalence of diabetes is particularly high in India and other low- and middle-income countries [2]. Treatment of diabetes involves a comprehensive lifestyle modification including dietary changes, increased physical activity, monitoring of blood sugar levels, regular intake of medications, self-care practices such as foot care and tooth care and reduction of risky behaviors like smoking and alcohol consumption. Therefore, diabetes self-management education is an essential component of treatment of patients [3].

Persons with disabilities are at a higher risk for type 2 diabetes as they have a lower level of physical activity and higher risk for obesity due to their restricted movement patterns [4]. Persons with disabilities are also particularly vulnerable to the harmful effects of diabetes as they have poorer access to health facilities, and poor treatment seeking behavior. Disabilities also are more common among the poor and underprivileged communities and therefore this adds a further layer of vulnerability. It is also possible that persons with disabilities find it more difficult to modify their lifestyles to adopt to the demands of diabetes treatment as many of them are dependent on their caregivers for even activities of daily living. Studies from several countries including a systematic review, have shown that there are extra costs associated with living with disabilities [5–7]. These extra costs make adoption of health lifestyles very difficult for persons with disabilities.

Rights of Persons with Disabilities Act 2016 lays emphasis on “…nondiscrimination, full and effective participation and inclusion in society, respect for difference and acceptance of disabilities as part of human diversity and humanity, equality of opportunity, accessibility, equality between men and women, respect for the evolving capacities of children with disabilities, and respect for the right of children with disabilities to preserve their identities. The principle reflects a paradigm shift in thinking about disability from a social welfare concern to a human rights issue.” [8]. Though this act has several provisions for persons with disabilities, and articulates these provisions as human rights, much remains to be achieved in this front.

The National Program for prevention and control of Cancer, Diabetes, Cardiovascular diseases, and Stroke (NPCDCS) was launched in the year 2010 with a focus on strengthening the infrastructure, human resources, and service delivery for these chronic non communicable diseases in India. Under this program drugs for type 2 diabetes are made available at the level of Primary Health Centre (PHC), the basic health facility at the village level [9].

However, the NPCDCS does not specifically have any interventions for the care of persons with disabilities who have diabetes. Blood tests for monitoring blood sugars and dispensing of medicines happens at the PHC level and persons with disabilities have barriers in accessing these PHCs. This leaves persons with disabilities who have diabetes out of the safety net created by the NPCDCS. In August 2021, the Government of Tamil Nadu, a southern state in India, has launched a new program called “Makkalai Thedi Maruthuvam” approximately translated to mean “Medicine at people’s doorstep”. According to this scheme, medicines for non-communicable diseases like type 2 diabetes, are dispensed at the doorstep of people with the disease, especially for the elderly and those with restricted mobility. This is likely to greatly enhance the access to these health services to persons with disabilities [10].

Primary Health Care is delivered at the grassroots level by the community health workers, namely the Auxiliary Nurse Midwives (ANM), Accredited Social Health Activists (ASHA), Anganwadi Workers (AWW) in India. They are part of the primary health care team which also comprises of doctors, nurses, physiotherapists, pharmacists, and laboratory technicians [11]. These primary health care providers are the face of the public health system, and they are responsible for delivery of primary diabetes care in the community. It is these primary health care providers who handle the challenges of treating persons in the community with disabilities who have diabetes. Understanding their experiences and the difficulties they face in delivering primary diabetes care to persons with disabilities will be very instructive in devising diabetes care policies for persons with disabilities. This qualitative exploration was conducted to explore the experiences of delivering diabetes care to persons with disabilities among these primary health care providers through in-depth interviews.

## Methods

### Qualitative approach and paradigm

We adopted a descriptive qualitative research approach with an objective of understanding the complex interplay of biological, social, environmental factors that influence the experiences of primary health care delivery for diabetes in persons with disabilities. Our research paradigm was social constructivist, ontological position was relativist, and epistemological stand was subjective. The assumption was that the reality of challenges and experiences of providing primary diabetes care for persons with disabilities is one which is socially constructed and subjective.

### Theoretical underpinning

Yeo and Moore have described the vicious cycle of interrelatedness between poverty and disability [12]. Persons with disability are excluded from education and employment opportunities, they have limited social capital, have poor access to health care and are low priority for all government interventions. This worsens their general state of health and wellness and pushes them into poverty. Chronic poverty worsens the disability and the exclusion from the society and exacerbates the disability. We used this theoretical model to inform our exploration. Informed by this theory, we developed a theoretical model to fit our research question. Persons with disabilities will first need to access the health facility to receive diabetes care. There may be challenges in reaching the facilities. After reaching the facilities, the health care providers give them drugs and life style modification advice. There may be challenges in providing regular medications and counseling about life style modifications. After providing these treatments, the health care providers may have to provide adequate support to help these persons adopt and maintain these treatments. There may be challenges in providing this supportive environment. We used this theoretical framework to guide our exploration.

### Researcher characteristics and reflexivity

The interviews were conducted by RT and VG. RT is a public health practitioner and researcher and is a physiotherapist by training. VG is a public health physician and researcher and is a medical doctor by training. VG also runs a diabetes clinic in a rural area through a voluntary health organization. The experiences of VG in providing diabetes care for persons with disabilities is likely to have influenced the interviews, analysis, and interpretation. RT and HS do not have direct experience of caring for persons with disabilities and hence their perspectives bring a different dimension to the analysis.

### Context of the study

The study was conducted among primary health care providers of the public health system of Tamil Nadu, a southern state in India. Tamil Nadu has a robust public health system with some of the best indicators of health. The prevalence of type 2 diabetes was also found to be high at 15% in Tamil Nadu. In Tamil Nadu still many public transport facilities like buses and trains remain inaccessible to persons with disabilities [13]. Public health facilities are also largely inaccessible to persons with disabilities [14]. These factors make health care access challenging for persons with disabilities in Tamil Nadu. The Makkalai Thedi Maruthuvam (MTM) scheme which has been launched in August 2021, is a unique scheme in Tamil Nadu which takes medications and testing facilities to the doorstep, especially for the elderly and those with restricted mobility.

### Sampling

To gain a sound understanding of primary health care providers’ experiences we sampled 6 primary health center medical officers, four primary health center nurses, one community physiotherapist and two village health nurses who delivered door to door services in the villages. We first approached the medical officers of the primary health center for the interviews, got their permission and then got the contact details of other community health workers in their team. We ensured that we sampled participants who were articulate and clear in expressing their opinions. We conducted 10 interviews initially and started perceiving that the same ideas were emerging in the interviews. We interviewed 3 more medical officers to confirm the emergence of data saturation and then concluded the interviews. Data saturation is defined as the state when subsequent interviews give the same ideas and themes without any new themes emerging [15].

### Ethical considerations

The study was reviewed by the Institutional Ethics Committee of ESIC Medical College and PGIMSR, KK Nagar, Chennai, India and approved with approval no. IEC/2021/1/21. The study was carried out as per the National Ethical Guidelines for Biomedical and Health Research involving Human Participants proposed by the Indian Council of Medical Research in 2017. Verbal informed consent was obtained from all participants prior to the interviews. The study was conducted during the second wave of the COVID 19 pandemic in India and hence many interviews were conducted through mobile phone or video calls. The process of verbal consent was approved by the Institutional Ethics Committee as it was felt that an audio-recorded verbal consent would be feasible and sufficient during a telephonic interview. The interviews were conducted on private phone lines or in a separate room and thus privacy was ensured. The confidentiality of the participants was protected by storing the recordings and transcripts in password protected files.

### Data collection methods

All interviews were conducted in the local language Tamil. We used a semi-structured interview checklist. This checklist is provided as Supplementary Material 1. While the checklist was used as a rough guide, we allowed the interviews to take a free flow and did not strictly adhere to it. We started the interview by asking whether they provide care for diabetes for persons with disabilities in their area. Then we asked about infrastructural challenges such as access issues, and disability inclusive spaces in the health facility. We then asked them about delivery of services like testing, dispensing medications, counseling for lifestyle modifications and follow up. Finally, we concluded by asking about specific challenges faced in delivering diabetes care to persons with disabilities. We obtained their permission and audio recorded their interviews. Where they did not provide consent for recording, we took notes of the interview. We transcribed all the interviews verbatim on the same day of the interview. We then translated the interview transcripts to English for ease of analysis. The interviews lasted from 30 min to 1 h. There was no compensation or incentives for the participants. Wherever feasible both RT and VG were present during the interview and we both discussed the contents of the interview to triangulate our understanding of the interview.

### Data analysis

Both VG and RT were involved in data analysis. We adopted Charmaz’s Grounded Theory approach principles to analyze the data, including concurrent collection and analysis of data, theoretical sampling, constant comparative approach to coding and a focus on theoretical saturation [16]. The transcripts were read even while data collection was progressing to familiarize with emerging theory and guide further interviews. However, a detailed coding was not done at this stage. After completing the interviews, VG initially read all the transcripts in detail several times and familiarized himself with the data. He performed open coding of the key statements in the transcripts. This was reviewed by RT. Where VG and RT disagreed, it was resolved by consensus or by arbitration by HS. After the first round of open coding of the 13 transcripts, the second round of analysis involved axial coding and theme building. We linked similar sounding codes along the same axis. For example, we linked all the codes that pertained to accessibility of the primary health center. We then looked at the emergent themes closely to understand the theoretical idea of challenges faced by health care providers in delivering primary diabetes care to persons with disabilities. We used the constant comparative approach at each level of coding by asking the question “How is this code related to the other codes in this axis?”. By using this method, we established linkages between various data points and developed a comprehensive theoretical understanding.

### Rigor of the research

We conducted interviews of primary diabetes care providers of different cadres, doctors, nurses, community health workers, who worked at the facility level as well as the community level. We also ensured that we interviewed providers in different districts with different levels of health system performance. There were interviews of providers from Chennai, Tirunelveli, Vellore which are well performing districts as well as Chengalpet, Villupuram, Tiruvallur, Tiruvannamalai, which are less well performing districts. This helped us obtain a good amount of transferability of the findings. At the end of each interview, we summarized our notes to the participants to ensure that we captured the information accurately. This helped build credibility of our findings. We also maintained detailed notes of all the interview processes. This helped establish an audit trail for ensuring dependability. To ensure confirmability, we maintained a reflexivity journal and referred to it constantly during analysis. Further, we achieved triangulation of our analysis by involving all three researchers in the data analysis.

## Results

All the primary health care providers who were interviewed had encountered and provided primary diabetes care for persons with disabilities. The participants were six medical officers (four men and two women), four staff nurses (all women), one physiotherapist (man) and two village health nurses (Auxilliary Nurse Midwife, both women). They belonged to the districts of Villupuram, Vellore, Tirunelveli, Thoothukudi, Thiruvallur, Chennai, Thiruvannamalai and Chengalpet. Other than one medical officer from urban Chennai and one medical officer from a remote tribal area in Vellore, all others were working in rural areas. All their interviews were rich with descriptions of their personal experiences, challenges faced and strategies adopted to overcome the challenges. As the number of interviews are very few, identifying who said which quote would make the quotes traceable to the person. For example, if we identified a quote as said by “Medical Officer in District A”, it may become easy to find out who said what. This can be politically sensitive, especially as these people are public health officials. So we have not identidied the demographic details of those who said these quotes in order to protect their confidentiality.

### Challenges in accessing the health facility

The primary health care facility was inaccessible to persons with disabilities. While most PHCs are in places which are reasonably well connected by public bus facilities and road facilities in Tamil Nadu, this level of access is often not adequate for persons with disabilities. Public transport itself is not very accessible to persons with disabilities. One of the community health workers said,

*“Our center is accessible by road. We have regular buses plying to the center. But persons with disabilities cannot even get into the bus. They must come by private autorickshaw. It becomes very expensive”*

Many PHCs lack ramp facilities and are not accessible by wheelchair. Even if the facility is accessible by wheelchair, the toilets are not. Newly constructed buildings usually have disability accessible features, but the older buildings are not accessible. One staff nurse mentioned,

*“Our PHC building does not have wheelchair access. This is because it is an old building. Such wheelchair access is available only at the nearest district hospital. So many persons with disabilities prefer to go to the district hospital than the PHC.”*

Many persons with disabilities preferred to use private hospitals and laboratories for their treatment and testing because they are closer to their homes and are more accessible. They preferred to use them despite having to pay a heavy price. One of the medical officers said,

*“Persons with disabilities prefer to use private labs and hospitals near their home even if it costs them money. This is because reaching this PHC can be difficult to them”*

Due to these challenges in accessibility, persons with disabilities are unable to come to the health facility. The caregivers are the ones who come and take the drugs from the PHC. Many such patients continue taking medications without consulting a physician or getting their blood tested.

### Challenges in giving medicines

Diabetes medicines are dispensed regularly for persons with disabilities. However, persons with disabilities are often on many medicines as they have multiple health problems. This results in them being confused about what medicines to take and how. One of the staff nurses mentioned,

*“Many times there is risk of too many drugs. They are on medicines for diabetes, high blood pressure, cholesterol, heart disease and in addition some vitamins also. They get confused about which medicines to take.”*

Some patients may have to visit different health facilities to get different medicines. Some medicines are available at the PHC as they are primary level drugs, whereas others such as cardiac drugs may only be available at the district level health facilities. This may lead to them missing some of the medicines. This is particularly problematic for persons with disabilities as they cannot move back and forth to different health facilities to get their medicines. One community health worker said,

*“We don’t give all the medicines here all the time. Only some drugs are available here. Nifedipine is not available here. Aspirin is not available sometimes. Stock is not available. So, we refer them to the district hospital to get those medicines. This is a great problem for persons with disabilities”*

Usually, these patients have poor compliance to medications. This is partly because of the irregular access to medications, dependence on others to obtain a refill of their prescription and poor awareness about the right way of taking the medicines. One of the medical officers said,

*“Treatment adherence is a challenge. This is because, they need a mobility support. One person should accompany them to the health facility since they cannot come by themselves. So, they don’t take medicines regularly.”*

During the COVID 19 times, due to lock down as well as compromise in delivery of NCD services due to prioritization of COVID 19 services, this access to medications was even more compromised.

### Challenges in lifestyle modifications

The greatest challenge in providing primary diabetes care for persons with disabilities is helping them adopt a healthy lifestyle. Restricted mobility precludes many physical activities. Following a healthy diet is also a challenge for persons with disabilities who are often poor. One of the medical officers said,

*“Lifestyle modification is a huge challenge for persons with disabilities. Medicines is all that I can sometimes offer them.”*

Persons with disabilities often experience intersecting dimensions of poverty, age and gender discrimination. They suffer from minor as well as major mental illnesses. Depression and anxiety lead to poor levels of motivation to adopt healthy lifestyles. Poor persons in rural areas with disabilities face neglect and discrimination at home due to their disability. So they are unable to follow a healthy diet. A staff nurse said,

*“But those who are disabled, they can only eat what they get. For some, even that is given in measured quantities. They (family members) say “You should only eat what I give”. They feel mentally upset because of this.”*

Primary care providers find it challenging to advice any kind of lifestyle modification for persons with disabilities as there are no specific guidelines.

### Challenges in adopting preventive measures

Persons with disabilities have poor follow up. They have poor access to testing for complications. All the tests for screening for complications are not available under one roof. While some PHCs have an ophthalmologist and a dentist visiting, most PHCs do not have these facilities and hence must refer to the higher center for screening for complications. One of the medical officers said,

*“When we refer to district hospital to get screened for secondary complications, only few people go. The others are unable to access the facility.”*

Another medical officer said,

*“If you want to get an opinion from various specialists to screen for complications, all these facilities are not under one roof. That’s the reason they don’t want to go to different places for testing. We haven’t customized it. If diabetes treatment is to be provided, foot care, wound care, podiatric center, ophthalmology center all should be in one place. But it is not so. How do you expect persons with disabilities to get these tests? This is a major limitation.”*

### Social challenges in providing diabetes care for persons with disabilities

Diabetes is a social disease in that a large part of its treatment depends on social interactions of the patient. Persons with disabilities have challenges in their social interactions. They are often viewed as non-productive and neglected by the society. The social neglect and family neglect has a negative impact on their diabetes care. Lack of family support is a major reason for poor outcomes of treatment of persons with disabilities. One of the medical officers said,

*“…there is lack of family support and specifically, there is no family support for elderly differently abled people”*

The level of family support also depends on the socio-economic status of the family. One of the medical officers said,

*“Family support is an issue. Family support is less. It depends upon the socio-economic status of the family. If they are well to do, they support them (persons with disabilities) well and take care of their needs. But if they are poor, family support is an issue.”*

### Local innovations by health care providers to overcome challenges

Primary health care providers adopt several local innovations and strategies to overcome the challenges of providing diabetes care for persons with disabilities. They collaborate with local non-governmental voluntary organization in helping the patients obtain disability aid, crutches, wheelchairs, and other devices that help them with carrying out their daily activities. One of the staff nurses said,

*“Local NGOs give disability aids. We coordinate with them to help our patients with disabilities. This helps them a little bit and this encourages them to continue their diabetes treatment.”*

Sometimes the health care providers utilize the outreach program funding of other programs to reach out to persons with disabilities. One of the medical officers mentioned that she used the Rashtriya Bal Swasthya Karyakram (RBSK), a program for outreach of children for screening and treatment of common illnesses, to reach out to persons with disabilities who live near the home of a child who is a beneficiary of the RBSK scheme. Such cross utilization of resources helped them reach out to persons with disabilities, even though there is no specific outreach program for them. One of the community health workers said,

*“We encourage the persons with disabilities to participate in vocational training classes organized by local NGO. This will help them develop some skills for earning a livelihood. It will help them continue their diabetes treatment”*

One of the staff nurses listed some innovative physical activities for persons who have visual and auditory disability. She said,

*“We ask them to walk along a figure of 8 in their own homes. This gives them some physical activity. We also ask those with limb disabilities to move their hands and legs even in a seated posture. This way we think they get at least some exercise”*

One medical officer reported having organized a peer support group of persons with diabetes. In this support group the able-bodied younger members provided support to the elderly and persons with disabilities. This model helped utilize the sense of solidarity within the community group to obtain social support for persons with disabilities. The medical officer said,

*“We have a patient support group for patients who are above 25 years of age. The support group meetings are happening from the last 3 months. They have asked us to organize in the 3 health sub-centers only, but we are doing it in all the 5 health sub centers under our facility. In the meetings, we stress the importance of screening for diabetes and hypertension especially for people who are 18 years and above. We also discuss and stress the importance of follow up, treatment adherence and regarding the complications of diabetes & high BP. The younger and physically stronger members of the group support the older and the disabled.”*

### Suggestions by the health care providers to improve diabetes care for persons with disabilities

The primary health care providers suggested that specific guidelines for diet and exercise be developed for diabetic patients living with disabilities. They also suggested that simple information, education, communication material be developed to distribute to persons with disabilities as well as their families. With increasing numbers of persons with disabilities this will be greatly helpful in improving their care. One of the medical officers said,

“We don’t have pamphlets or posters exclusively for them (persons with disabilities). We have general IECs on diet which we provide it to them and counsel. But making them to do a physical activity is a challenge. If they can walk, we advise walking. We need to develop unique guidelines for them and must also make IEC materials specific to them.”

Incentivizing the regular intake of medicines, adoption of lifestyle modifications and regular testing and visits would encourage persons with disabilities to adopt these behaviors. These could be in the form of fruit and vegetable vouchers or even cash incentives. The cash incentive itself will motivate family members to respect and support the diabetes care of persons with disabilities. One medical officer said,

“We should incentivize the health services for differently abled people. Atleast then their care givers might be interested in showing some interest in their health. Like how Rs. 500 is given for patients with TB, some amount should be provided to these people for availing diabetes services. This is because, then the care takers might show interest in the incentive and in turn they will bring differently abled people for health checkups.”

The health care providers also suggested that there should be coordination between the health and social welfare departments, so that persons with disabilities are covered by both the welfare schemes and the health schemes. One community health worker said,

“Disability aids, wheelchairs, three-wheeler bikes etc. are under the department of social welfare. They only maintain the detailed database of persons with disabilities. We only handle diabetes related services and health care services. There should be sharing of data and coordination of services between these departments to improve the care.”

## Discussion

In this qualitative study of challenges faced by primary health care providers in delivering diabetes care for persons with disabilities we found that the patients found it difficult to access the primary health centers which were not disability friendly. Often medications were the only treatment provided for these patients as they could not follow any lifestyle modifications due to their disabilities. Even these medications were often obtained by the care givers from the health facilities without monitoring the blood sugar controls. Monitoring of the blood sugar levels is essential for effective treatment of diabetes. But this was not available for persons with disabilities. This is a violation of their basic right to good quality treatment. The COVID 19 pandemic further hampered the access to these medicines. Low mood, depression, anxiety led to decreased levels of motivation that prevented adoption of health lifestyles. Due to restricted mobility these patients also did not undergo periodic eyes, dental and other checkup to screen for complications of diabetes. There was lack of family support, especially for the elderly and the poor with disabilities, which prevented proper diabetes care. Some primary health care providers did some local innovations to overcome these challenges including cross utilization of resources from other programs to reach out to persons with disabilities, formation of peer support groups and identification of innovative physical activities that even persons with disabilities can perform. The primary health care gave some suggestions to improve diabetes care for persons with disabilities including development of specific information, education, and communication materials, incentivizing visits to health facilities and adoption of health behaviors and intersectoral coordination between social welfare department and health department. In this paragraph we shall discuss these points in detail.

Studies from rural Malawi and South Africa showed that persons with disabilities find it difficult to access health care services due to cost of transport, insufficient health care resource availability, and dependence on others [17, 18]. In a study from south India, it was seen that people with disabilities had a greater burden of type 2 diabetes, but also suffered significantly greater barriers to accessing health care services for diabetes [19]. As seen earlier, persons with disabilities are caught in a vicious cycle of poverty and exclusion. This impedes their access to health care and care for diabetes [12]. Persons with disabilities not only face physical barriers to accessing health care services, but they also face discrimination and stigmatization in the health facilities, which further limits their access [20]. Similar limitations in access to health facilities was reported by the primary health care providers in this study. The providers mentioned that persons with disabilities do not visit their primary health center regularly because the public transport was not accessible. So most of the patients with diabetes only took their prescription refills by proxy through their care givers.

Tamil Nadu is one of the states in India with an excellent logistics and supply chain of medications at all levels of care [21]. However, all drugs are not available at all levels of the public health system. The medication available at a particular level of health care is appropriate to the essential drug list for that level of care. Therefore, some cardiac medications and higher order drugs may not be available at primary health care level. This makes patients go back and forth between various hospitals to get their full set of medications. Persons with disabilities find this particularly difficult. A study of adherence to anti-hypertensive medications among persons with disabilities showed that they had poorer adherence compared to those without disabilities [22]. In a study of medical adherence among patients with rheumatic diseases, it was found that disabilities greatly reduced the adherence [23]. In another study from Shanghai, it was seen that physical disabilities greatly reduced adherence to medications among patients with chronic conditions [24]. Medications may be the only form of diabetes treatment for persons with disabilities, and this too has a poor adherence. Therefore, the treatment that these patients receive for their diabetes is grossly inadequate. During the COVID 19 times, even the medications were inaccessible to many persons with disabilities, due to the lockdown restrictions and lower prioritization of chronic non communication diseases in comparison to COVID 19 [25].

Poverty, illiteracy, gender, and age produced an intersectional complex vulnerability to persons with disabilities in preventing them from obtaining adequate care for their diabetes [26, 27]. The dependency of persons with disabilities on others was particularly problematic when these others were the sole breadwinners and could not spare the time to care for them. This was worsened if the person with disability was a woman and was older because she was seen as less valuable than other able-bodied counterparts. Adoption of healthy lifestyles including a healthy diet, physical activity were more expensive affairs and the complex intersection of poverty, gender, age, and disability precluded persons with disabilities from adopting healthy lifestyles. While specific interventions exist in the US and UK for persons living with disabilities to adopt healthy lifestyles, such interventions do not exist in India [28]. Lack of such lifestyle modification guidelines for persons with disabilities prevents primary care providers from giving them any specific advice. Not only this, the persons with disabilities are also unable to undergo periodic screening for complications of diabetes such as ophthalmologic screening, dental screening, heart screening etc. This greatly compromises the quality of primary diabetes care that these patients receive.

Family support is an essential component of diabetes care. Previous studies have shown that good amount of family support is required for adequate glycemic controls [29, 30]. This becomes even more important for persons with disabilities as they are dependent on their family members for even their daily activities. However, many persons with disabilities do not receive adequate family support. The receipt of family support is worse for those from poorer families.

The Rights of Persons with Disabilities Act of 2016, as seen earlier creates a paradigm shift from a social welfare approach to a rights-based approach to care for persons with disabilities [8]. This approach would mean that persons with disabilities have a basic right to good health and wellbeing. This would also include right to care for diabetes and right to protection from complications of diabetes.

The interviews highlighted the difficulties and challenges faced by the primary health care providers in delivering diabetes services to persons with disabilities. The new Makkalai Thedi Maruthuvam (Medicine at people’s doorstep) scheme of the government of Tamil Nadu, which took diabetes medications and testing services to the doorsteps of persons with disabilities was eagerly welcome by these providers. Some of these primary care providers innovated and adopted strategies to overcome challenges of caring for these patients. These strategies, namely formation of peer support group, innovative brief physical activities and linking of outreach activities of other programs to reach out to persons with disabilities are worth studying closely and integrating into the regular policy of the state. There are some limitations of this study including a small sample size. However, the problems of treating persons with disabilities is universal and the experiences are common, that even this small sample size was able to provide us adequate data saturation. We did not interview persons with disabilities as we mainly intended the capture the supply side challenges. A more detailed exploration of lived experiences of persons with disabilities must be done in the future to complement the findings of this study.

## Conclusions

Primary health care providers faced several challenges in delivering primary diabetes care for persons with disabilities both at the facility level and at the community level. They also developed their own innovative strategies to overcome these challenges. There is a need to develop a strong health policy addressing the need for chronic diabetes care for persons with disabilities that is accessible, of good quality and equitable.

## Supplementary Information

**Additional file 1.**

## Data Availability

The data is available with the corresponding author on reasonable request through email. Most of the verbatim quotes are provided in the results section.

## Abbreviations

ANM: Auxilliary Nurse Midwife
ASHA: Accredited Social Health Activist
AWW: Anganwadi Worker
IEC: Information Education Communication
MTM: Makkalai Thedi Maruthuvam
NCD: Non Communicable Diseases
NGO: Non Governmental Organization
NPCDCS: National Program for Control of Cancer, Diabetes, Cardiovascular Diseases and Stroke
PHC: Primary Health Centre
RBSK: Rashtriya Bal Swasthya Karyakram
TB: Tuberculosis

## References

[1] Khan MAB, Hashim MJ, King JK, Govender RD, Mustafa H, al Kaabi J (2020). Epidemiology of Type 2 Diabetes - Global Burden of Disease and Forecasted Trends. J Epidemiol Glob Health.

[2] Pradeepa R, Mohan V (2021). Epidemiology of type 2 diabetes in India. Indian J Ophthalmol.

[3] Chrvala CA, Sherr D, Lipman RD (2016). Diabetes self-management education for adults with type 2 diabetes mellitus: a systematic review of the effect on glycemic control. Patient Educ Couns.

[4] McDermott S, Moran R, Platt T, Dasari S (2007). Prevalence of diabetes in persons with disabilities in primary care. J Dev Phys Disabil.

[5] Parey B, Sinanan L (2022). Healthcare barriers among working-age persons with disabilities in Trinidad. Qual Health Res.

[6] Mitra S, Palmer M, Kim H, Mont D, Groce N (2017). Extra costs of living with a disability: a review and agenda for research. Disabil Health J.

[7] Morris ZA, Zaidi A (2020). Estimating the extra costs of disability in European countries: implications for poverty measurement and disability-related decommodification. J Eur Soc Policy.

[8] Narayan C, John T (2017). The Rights of Persons with Disabilities Act, 2016: does it address the needs of the persons with mental illness and their families. Indian J Psychiatry.

[9] Thakur JS, Paika R, Singh S (2020). Burden of noncommunicable diseases and implementation challenges of National NCD Programmes in India. Med J Armed Forces India.

[10] Special Correspondent. TN CM launches Makkalai Thedi Maruthuvam scheme. Chennai: The Hindu; 2021.

[11] Scott K, George AS, Ved RR (2019). Taking stock of 10 years of published research on the ASHA programme: examining India’s national community health worker programme from a health systems perspective. Health Res Policy Syst.

[12] Yeo R, Moore K (2003). Including disabled people in poverty reduction work: “Nothing About Us, Without Us”. World Dev.

[13] Srikanth R, Poorvaja S. Accessibility in public transport still elusive for disabled persons. Chennai: The Hindu; 2021.

[14] Sharma A. Persons with disabilities face double whammy of inaccessibility and official apathy in the pandemic. Chennai: The Hindu; 2021.

[15] Saunders B, Sim J, Kingstone T, Baker S, Waterfield J, Bartlam B (2018). Saturation in qualitative research: exploring its conceptualization and operationalization. Qual Quant.

[16] Charmaz K (2014). Constructing Grounded Theory.

[17] Vergunst R, Swartz L, Hem KG, Eide AH, Mannan H, MacLachlan M (2017). Access to health care for persons with disabilities in rural South Africa. BMC Health Serv Res.

[18] Harrison JAK, Thomson R, Banda HT, Mbera GB, Gregorius S, Stenberg B (2020). Access to health care for people with disabilities in rural Malawi: what are the barriers?. BMC Public Health.

[19] Gudlavalleti MVS, John N, Allagh K, Sagar J, Kamalakannan S, Ramachandra SS (2014). Access to health care and employment status of people with disabilities in South India, the SIDE (South India Disability Evidence) study. BMC Public Health.

[20] Senjam S, Singh A (2020). Addressing the health needs of people with disabilities in India. Indian J Public Health.

[21] Tatambhotla A, Choksi M, Singh PV, Kalvakuntla RR (2012). Replicating Tamil Nadu’s drug procurement model. Econ Polit Wkly..

[22] Park JH, Park JH, Lee SY, Kim SY, Shin Y, Kim SY (2008). Disparities in antihypertensive medication adherence in persons with disabilities and without disabilities: results of a Korean Population-Based Study. Arch Phys Med Rehabil.

[23] Goh H, Kwan YH, Seah Y, Low LL, Fong W, Thumboo J (2017). A systematic review of the barriers affecting medication adherence in patients with rheumatic diseases. Rheumatol Int.

[24] Huang J, Jiang Z, Zhang T, Wang L, Chu Y, Shen M, et al. Which matters more for medication adherence among disabled people in Shanghai, China: family support or primary health care? Inquiry. 2019;56:46958019883175. 10.1177/0046958019883175 PMC6804357 31631723

[25] Gummidi B, John O, Jha V (2020). Continuum of care for non-communicable diseases during COVID-19 pandemic in rural India: a mixed methods study. J Family Med Prim Care.

[26] Pinilla-Roncancio M (2015). Disability and poverty: two related conditions. A review of the literature. Revista de la Facultad de Medicina..

[27] Banks LM, Kuper H, Polack S (2017). Poverty and disability in low- and middle-income countries: a systematic review. PLoS One.

[28] Centres for Disease Control and Prevention (2020). Disability and Diabetes Prevention. Atlanta.

[29] Mayberry LS, Osborn CY (2012). Family support, medication adherence, and glycemic control among adults with type 2 diabetes. Diabetes Care.

[30] Ravi S, Kumar S, Gopichandran V (2018). Do supportive family behaviors promote diabetes self-management in resource limited urban settings? A cross sectional study. BMC Public Health.

