# Supplementary material for: “Medicines is all that I can sometimes offer them”: challenges of providing primary diabetes care to persons with disabilities in Tamil Nadu
Source: BMC Health Serv Res. 2022 Jul 5;22:861. doi: 10.1186/s12913-022-08246-1 (PMC9253259; doi:10.1186/s12913-022-08246-1)
Supplement: Supplementary file 1 — Additional file 1. [file 12913_2022_8246_MOESM1_ESM.docx]

**“Medicines is all that I can sometimes offer them”: Challenges of providing primary diabetes care to persons with disabilities**

**In Depth Interview Check List for Medical Officers**

Welcome to this interview. Thank you for agreeing to participate. I am doing a research study on diabetes care services provided by the public health system for persons living with disabilities. I would like to interview you regarding this issue and gain an understanding of your experiences in this matter. Shall I record this interview?

1. Do you see patients with disabilities who have diabetes in your OPD?
2. On an average how commonly do you see patients with disabilities and diabetes? What about persons with mental disabilities?
3. If they respond not very commonly ask – why do you think so? Is it because PWD do not get diabetes? Or do you think they do not access health care? Could you explain?
4. If they respond frequently – do you think the burden of diabetes is higher among PWD? If yes, can you explain why?

**Infrastructural issues:**

1. Is your health facility disability friendly? Does it have ramp? Railings? Wheelchair accessible toilets? Corridors?
2. Does your facility have wheelchairs? Walking aids?

**NCD services**

1. Do you have outreach clinics for PWD? Does your MMU cover PWD?
2. Do you have database of PWD? Do you have database of PWD with diabetes?
3. Do you take any special measures to dispense medications to PWD with diabetes?
4. Do you have any follow up mechanism for PWD with diabetes?
5. Do you have diet counseling services?
6. Do you have physical exercise training? Do you have for PWD in particular?
7. Do you create awareness through IEC materials on LSM for diabetes among PWD?

**Challenges in treating diabetes among PWD**

1. What are some of the challenges that you face in treating PWD with diabetes?
2. Do you face challenges with drug adherence?
3. Do you face challenges with monitoring of blood parameters BP etc?
4. Do you face challenges with LSM advice? How do you teach exercise? How do you promote exercise? Are there disability friendly parks, public spaces where they can exercise?
5. Do you face challenges in screening for complications? Do you do annual blood exams for them?
6. How do you overcome these challenges?
7. What should government do to improve the quality of services provided to PWDs with diabetes?

**In Depth Interview Check List for Village Health Nurses, Nurses, Physiotherapist**

Welcome to this interview. Thank you for agreeing to participate. I am doing a research study on diabetes care services provided by the public health system for persons living with disabilities. I would like to interview you regarding this issue and gain an understanding of your experiences in this matter. Shall I record this interview?

1. Do you see patients with disabilities who have diabetes in your field area?
2. On an average how commonly do you see patients with disabilities and diabetes? What about persons with mental disabilities?
3. If they respond not very commonly ask – why do you think so? Is it because PWD do not get diabetes? Or do you think they do not inform you? Could you explain?

**Infrastructural issues:**

1. Is your health facility disability friendly? Does it have ramp? Railings? Wheelchair accessible toilets? Corridors?
2. Does your facility have wheelchairs? Walking aids?

**NCD services**

1. Do you have outreach clinics for PWD? Do you do door to door visits?
2. Do you have database of PWD? Do you have database of PWD with diabetes?
3. Do you take any special measures to dispense medications to PWD with diabetes?
4. Do you have any follow up mechanism for PWD with diabetes?
5. Do you have diet counseling services?
6. Do you have physical exercise training? Do you have for PWD in particular?
7. Do you create awareness through IEC materials on LSM for diabetes among PWD?

**Challenges in treating diabetes among PWD**

1. What are some of the challenges that you face in providing care for PWD with diabetes?
2. Do you face challenges with drug adherence?
3. Do you face challenges with monitoring of blood parameters BP etc?
4. Do you face challenges with LSM advice? How do you teach exercise? How do you promote exercise? Are there disability friendly parks, public spaces where they can exercise?
5. Do you face challenges in screening for complications? Do you do annual blood exams for them?
6. How do you overcome these challenges?
7. What should government do to improve the quality of services provided to PWDs with diabetes?
